# Ethical Challenges and Implications of Telemedicine Implementation in Saudi Primary Healthcare: A Narrative Review

**DOI:** 10.7759/cureus.100041

**Published:** 2025-12-24

**Authors:** Bandar S Alzuair, Waleed A Al Mufarrih, Ashwag A Alzahrani, Mohammed S Almasri

**Affiliations:** 1 Family Medicine, King Salman Armed Forces Hospital, Tabuk, SAU; 2 Microbiology, King Salman Armed Forces Hospital, Tabuk, SAU; 3 Radiology Department, King Saud Medical City, Riyadh, SAU

**Keywords:** health equity, primary health care, privacy, saudi arabia, telemedicine

## Abstract

Telemedicine has become a central component of Saudi Arabia’s ongoing digital health transformation, offering new opportunities to enhance accessibility, efficiency, and patient-centered care. As virtual services expand, ethical considerations have grown increasingly important, particularly regarding informed consent, privacy and data security, professional boundaries, and equitable access for vulnerable populations. This narrative review synthesizes evidence from empirical studies, policy analyses, and ethical frameworks to examine the key ethical challenges associated with telemedicine in Saudi primary care. Findings highlight significant variations in digital literacy, persistent disparities among older adults and low-income groups, and increasing physician workload associated with virtual communication demands. Cultural norms surrounding family involvement further complicate consent and disclosure processes, underscoring the need for context-specific guidance. The review emphasizes that safe and equitable telemedicine implementation requires clear ethical protocols, culturally aligned decision-making practices, improved digital health infrastructure, and institutional support for clinicians. Strengthening governance, standardizing virtual care procedures, and integrating digital health equity principles are essential for sustaining high-quality telemedicine services. Overall, telemedicine holds substantial potential to advance healthcare delivery in Saudi Arabia, provided that ethical, cultural, and operational challenges are addressed through coordinated clinical and policy efforts.

## Introduction and background

Technological transformation has become a defining feature of modern healthcare systems, with telemedicine emerging as a central component of digital health strategies worldwide. In Saudi Arabia, national reforms under Vision 2030 have prioritized digitalization as a key mechanism to enhance accessibility, efficiency, and quality of care across the health sector [1]. Recent policy-oriented and narrative evaluations of Saudi digital health initiatives describe notable progress in national digital infrastructure, health information interoperability frameworks, and institutional preparedness for technology-enabled care, suggesting an increasingly supportive environment for the expansion of virtual care services [1,2].

Telemedicine adoption in Saudi Arabia accelerated rapidly during the COVID-19 pandemic, supported by national guidance, institutional readiness, and increased clinical demand [3,4]. Cross-sectional studies conducted primarily in major urban centers, including Riyadh, report generally favorable physician attitudes toward telemedicine, with respondents describing perceived usefulness in routine practice and benefits in flexibility and continuity of care; however, these findings are derived from limited samples and may not fully reflect acceptance across all regions or practice settings in Saudi Arabia [3,5]. From the patient perspective, telemedicine has facilitated improved access to services, particularly during public health restrictions, although utilization remains variable across demographic groups [6,7]. Evidence from local and international literature suggests that, in selected clinical contexts, telemedicine is associated with improvements in patient satisfaction and care processes, and, in some settings, selected clinical outcomes, when appropriately integrated into care pathways, although the supporting evidence is variable and generally of low-to-moderate quality [8].

Despite these advances, telemedicine introduces complex ethical, legal, and organizational challenges. Global reviews consistently highlight concerns related to privacy, confidentiality, data security, informed consent, and clinician responsibility during virtual encounters [9,10]. Broader structural and political determinants, including technological infrastructure, socioeconomic disparities, and digital literacy, also influence equitable access to digital health services [11,12]. Without careful planning, these factors may contribute to variations in adoption, quality, and safety of telemedicine across different patient populations and care settings.

As Saudi Arabia continues to expand digital health services, particularly within primary care, existing literature remains fragmented, with ethical guidance often dispersed across regulatory documents, specialty-specific discussions, and international frameworks, and limited synthesis addressing culturally and operationally relevant ethical challenges; this underscores the need for a contextually grounded ethical framework to guide telemedicine practice. Understanding the interplay between technological capabilities, professional practices, sociocultural norms, and systemic structures is essential for ensuring that telemedicine supports safe, equitable, and trustworthy patient care. This narrative review synthesizes empirical evidence, ethical analyses, and regional contextual factors to outline key ethical considerations and practical implications for telemedicine implementation in Saudi primary healthcare.

## Review

Methodology

A narrative literature review was conducted to examine the ethical, legal, and cultural dimensions of telemedicine in primary healthcare, with a focus on Saudi Arabia and comparable healthcare contexts. Comprehensive searches were performed in PubMed, Scopus, Web of Science, and the World Health Organization Eastern Mediterranean Regional Office (EMRO) databases for studies published between January 2015 and October 2025, using a combination of controlled vocabulary and free-text terms. The search strategy incorporated Medical Subject Headings (MeSH) and keyword variants related to telemedicine and ethics. For PubMed, the following search string was applied: (“telemedicine”[MeSH] OR telemedicine OR telehealth OR “virtual care”) AND (“ethics”[MeSH] OR ethics OR bioethics OR “professional responsibility” OR “informed consent” OR privacy) AND (Saudi Arabia OR Saudi). Equivalent search terms and Boolean operators were adapted for other databases to account for platform-specific indexing systems.

Articles were eligible for inclusion if they: (1) addressed ethical, legal, or cultural dimensions of telemedicine; (2) focused on primary healthcare settings; (3) examined issues related to informed consent, privacy, confidentiality, or professional boundaries; (4) reported empirical studies, reviews, policy analyses, or clinical guidelines; (5) were published in English or Arabic; and (6) were relevant to Saudi Arabia or to healthcare systems with comparable cultural and ethical contexts, particularly within Middle Eastern or Gulf Cooperation Council (GCC) settings.

The screening process was conducted in two stages. After removal of duplicates, titles and abstracts were independently screened by two reviewers to assess preliminary eligibility based on the predefined criteria. Full-text screening was subsequently performed for articles meeting initial inclusion criteria. Disagreements regarding study inclusion were resolved through discussion and consensus. The initial search yielded approximately 2,847 records. Following title and abstract screening, 387 articles met preliminary eligibility criteria. Of these, 231 were excluded after full-text review for not meeting inclusion requirements, resulting in 156 articles that informed the thematic synthesis. Given the narrative nature of this review, not all included studies were individually cited; instead, representative studies were selected to support key ethical themes.

Data extraction captured bibliographic characteristics, study design, primary ethical focus, key findings, and geographic context. Extracted data were synthesized narratively and organized thematically around major ethical domains relevant to telemedicine practice, including informed consent, privacy and confidentiality, professional boundaries, and culturally contextual ethical considerations. Studies were excluded if they: (1) focused exclusively on technical infrastructure or clinical efficacy without ethical analysis; (2) consisted of opinion pieces or editorials lacking an evidence-based foundation; (3) examined highly specialized clinical applications without relevance to primary care ethics; (4) presented single-case reports without generalizable ethical insights; or (5) lacked applicability to Saudi Arabia or comparable regional contexts.

Telemedicine in the Saudi context

Saudi Arabia has prioritized digital health transformation as a central component of its national Vision 2030 strategy, aiming to improve healthcare accessibility, efficiency, and quality through large-scale integration of digital tools, virtual care platforms, and health informatics systems [1,2]. These national initiatives have accelerated the adoption of telemedicine across primary and secondary care settings, supported by investments in digital infrastructure, interoperability, and clinical readiness [1,2,13].

Empirical evidence indicates that healthcare practitioners in Saudi Arabia generally hold favorable attitudes toward telemedicine. Studies conducted among physicians in Riyadh demonstrate confidence in using telemedicine technologies, recognition of their value in routine clinical workflows, and acknowledgment of telemedicine’s utility in supporting flexibility, continuity of care, and timely patient communication [3,5]. These findings align with broader evaluations of clinician attitudes during the COVID-19 pandemic, when telemedicine became an essential mode of service delivery due to infection control measures and national-level guidance promoting remote care [3,5,7].

From the patient perspective, telemedicine has been associated with increased access to healthcare services, particularly during periods of restricted mobility and heightened system strain, such as the COVID-19 pandemic; however, evidence suggests that sustained patient utilization and engagement remain variable outside these acute contexts. The national 937 call center, for example, has been widely used by patients seeking advice, triage, and remote support, reflecting strong public engagement with virtual care options [7]. However, patient utilization remains uneven. A cross-sectional study among individuals with chronic conditions revealed that telemedicine use is significantly influenced by demographic and socioeconomic factors, including age, education level, and digital literacy [6]. Similarly, studies focusing on Saudi older adults highlight barriers to technological confidence, usability challenges, and difficulties with navigating health systems, which may limit uptake among aging populations [14,15].

Despite these disparities, international and regional evaluations indicate that telemedicine has the potential to enhance patient satisfaction, improve care processes, and expand access to healthcare, with evidence of improved clinical outcomes primarily documented in selected conditions (such as chronic disease monitoring and mental health follow-up), and largely derived from observational and short-term studies [8]. These findings underscore the importance of developing targeted strategies to address demographic and structural barriers, ensuring that the benefits of telemedicine extend equitably across Saudi populations as the healthcare system continues its digital transformation [6,13-15].

Informed consent and family-centered care

Informed consent in telemedicine introduces distinct ethical and practical challenges, as clinicians must ensure patient understanding and voluntariness without the contextual cues of in-person encounters. In Saudi Arabia, these challenges are shaped by cultural norms and Islamic ethical principles that influence disclosure practices and the role of families in healthcare decisions [16-18]. Islamic bioethics emphasizes balancing patient autonomy with compassionate family support, reflecting long-standing traditions in which relatives participate actively in treatment discussions, particularly in serious or sensitive medical situations [16,18].

Empirical studies from Saudi healthcare settings indicate that family involvement in care and decision-making is commonly observed and often valued, although the degree and nature of involvement vary depending on patient preferences, clinical context, and sociocultural factors. Many families assume a central role in treatment decisions, which can strengthen support systems but also complicate a physician’s duty to respect patient autonomy when preferences differ [18,19]. Research on shared decision-making shows that Saudi patients vary widely in their preferences: some favor family-inclusive collaboration, others lean toward autonomous decision-making, and some prefer a more paternalistic physician-led model [19]. Similar patterns across the Eastern Mediterranean Region highlight how sociocultural expectations shape communication dynamics and decision authority [20].

Telemedicine adds further complexity. Remote consultations make it difficult to verify who is present with the patient, whether disclosures are voluntary, and how much influence family members may be exerting. Patient-centered telemedicine frameworks, therefore, recommend explicitly asking patients about their disclosure preferences and the role they want family members to play, rather than assuming uniform cultural expectations [21]. This aligns with Saudi patient-preference models emphasizing individualized assessments of autonomy within cultural and religious contexts [18].

Digital informed consent tools offer potential benefits, such as improved clarity and standardized documentation, yet challenges remain related to digital literacy, usability, and variable comprehension. Evidence from randomized trials demonstrates that telehealth-based consent can achieve comprehension equivalent to in-person consent, sometimes with improved information retention [22]. Studies examining consent for digital technologies, such as medical AI, further show that patient trust, clarity of information, and transparency determine perceived consent adequacy more than the physical setting [23]. Practical obstacles, including limited health literacy or time constraints, may still impede understanding and can be amplified in remote settings [24]. An ethically robust telemedicine consent process in Saudi primary care, therefore, requires clear explanation of virtual care risks and limitations, verification of identity and capacity, explicit inquiry into family involvement preferences, and documentation that reflects individualized disclosure choices within an Islamic ethical framework [16-19,21].

Privacy, confidentiality, and data security

Privacy and confidentiality are among the most prominent ethical concerns in telemedicine, as remote care introduces risks related to unauthorized access, insecure communication channels, and variable control over data transmission and storage [9,10]. These risks are heightened by the widespread use of mobile devices and third-party platforms that may not consistently meet healthcare privacy standards [9.10]. Effective health information governance requires clear policies, standardized procedures, and strong oversight mechanisms to ensure transparency, integrity, and accountability across digital care systems [25]. Such frameworks are essential for building patient trust and supporting safe telemedicine expansion.

Global assessments of healthcare data protection identify persistent challenges, including inconsistent regulatory safeguards, cybersecurity vulnerabilities, and gaps in interoperability across digital platforms [26]. Patients frequently express concerns regarding confidentiality, surveillance, and potential secondary use or misuse of health data, concerns that directly influence their willingness to engage in telemedicine [26]. Systematic reviews of mobile health applications similarly show that Saudi patients often worry about data sharing, weak security protections, and uncertainty over who can access their personal information [27-29]. These findings highlight the need for transparent communication about privacy safeguards and user-centered design that clearly explains risks, permissions, and data flows.

Saudi Arabia’s digital transformation initiatives have increasingly prioritized cybersecurity in healthcare, focusing on strengthening information systems, enhancing cyber resilience, and protecting against unauthorized access as telemedicine adoption accelerates [30]. As virtual care platforms integrate artificial intelligence and advanced analytics, new vulnerabilities emerge, including algorithmic manipulation and system-level cyberattacks. Recent policy analyses recommend developing integrated cybersecurity strategies specifically tailored for AI-enabled healthcare systems [31]. Narrative reviews also suggest that blockchain and distributed ledger technologies may enhance security by improving traceability, integrity, and tamper resistance of medical data [32]. Overall, safeguarding privacy and confidentiality in telemedicine requires robust governance structures, culturally appropriate communication, clear regulatory guidance, and continuous monitoring of evolving digital threats. These elements are essential for maintaining patient trust and supporting sustainable telemedicine expansion in Saudi primary care.

Professional boundaries and vulnerable populations

Telemedicine creates new challenges in maintaining professional boundaries, as digital platforms blur distinctions between clinical and non-clinical communication. Remote care often increases the frequency and informality of interactions, making it harder for clinicians to manage expectations around availability and uphold consistent standards of conduct [9]. Implementation studies show that introducing digital tools can create uncertainty regarding roles, triage responsibilities, and workflow organization, especially when expectations for communication are not clearly defined [33]. Nursing literature further notes that digital care shifts clinical labor in ways that require training and coordination to ensure continuity and safety [34].

Maintaining boundaries is essential for patient safety. Quality assurance research highlights the importance of structured triage, standardized documentation, and verification procedures to prevent errors and ensure consistent care during virtual encounters [35]. Systematic reviews similarly stress the need for clear communication standards, reliable follow-up processes, and protocols to manage technical disruptions in remote settings [36]. Certain groups, such as individuals with limited digital literacy, cognitive impairment, language barriers, or complex medical needs, may be especially vulnerable in telemedicine. Studies indicate clinicians must apply additional effort to verify identity, assess capacity, and ensure comprehension when working with these populations [37]. Clear policies, clinician training, and structured communication strategies are therefore crucial to maintaining safe and ethical boundaries in virtual care.

Digital health equity

Digital health equity is a critical issue in the expansion of telemedicine, as disparities in digital access, literacy, and infrastructure can disproportionately affect vulnerable groups. Structural and political determinants, including socioeconomic conditions, connectivity distribution, and governance priorities, shape who can benefit from virtual care [11]. Scoping reviews show that inequalities in digital health are frequently driven by gaps in digital literacy, affordability, and access to technology [12].

In Saudi Arabia, telemedicine usage varies across demographic groups: younger, higher-income, and better-educated individuals are more likely to engage with digital services, while older adults, low-income populations, and rural residents exhibit lower use [6]. Globally, groups such as maternal and child health populations also face compounded barriers, including limited connectivity, linguistic challenges, and reduced familiarity with digital tools [38].

Digital health equity frameworks emphasize that equitable telemedicine requires more than technical availability. Systems must incorporate inclusive design, culturally aligned communication, and support mechanisms that address social and structural barriers [39]. Without such considerations, digital transformation can unintentionally widen health disparities due to unequal technological readiness and trust in digital systems [40]. Infrastructure gaps such as inadequate broadband and limited institutional support further disadvantage underserved communities [41,42]. Advancing equity in telemedicine requires targeted efforts to improve digital literacy, expand affordable internet access, and ensure culturally adapted platforms. Addressing structural determinants is essential for ensuring that virtual care supports, rather than undermines, fair and inclusive access [11-12,39-42].

Older adults and telemedicine barriers

Older adults face significant challenges in using telemedicine, despite the growing availability of digital health services in Saudi Arabia. Common barriers include limited experience with digital devices, difficulty navigating application interfaces, and lower confidence in relying on virtual care [14]. Research on mobile health use among older Saudi adults similarly shows interest in digital tools but highlights obstacles such as unfamiliarity with technology, vision problems, and difficulty understanding complex app features [15]. Broader telemedicine literature adds that older individuals often require additional support during virtual encounters, as digital unfamiliarity can lead to anxiety and reduced trust [34]. Global reviews also note reduced digital literacy, physical limitations, and privacy concerns as persistent barriers [43]. Addressing these issues requires simplified designs, tailored training, and caregiver involvement to ensure equitable access for older adults [14,15].

Integration of ethical pillars

Integrating telemedicine ethically requires applying the core biomedical principles of autonomy, beneficence, non-maleficence, and justice within Saudi Arabia’s cultural, religious, and technological context. Islamic bioethical scholarship emphasizes that these principles must be interpreted through foundational ethical values that prioritize the preservation of life, intellect, and human dignity [16,17]. This framework provides culturally grounded guidance for clinical decision-making in telemedicine, particularly regarding disclosure, patient empowerment, and family involvement.

Autonomy in Saudi Arabia is shaped by family-centered care norms, where relatives often participate in treatment discussions and influence decision-making [18]. Islamic ethical guidance acknowledges the legitimacy of family involvement but stresses that the patient’s own wishes should remain central [16,18]. Telemedicine systems must therefore incorporate mechanisms that allow clinicians to clarify patient preferences concerning disclosure and the desired role of family members, rather than relying on assumed cultural patterns. Beneficence and non-maleficence require that telemedicine promote patient well-being while minimizing risks. Patient-centered telemedicine frameworks highlight the importance of clear communication, identity verification, and assessing the clinical suitability of remote encounters [21]. These safeguards help ensure that telemedicine improves access and continuity without compromising diagnostic accuracy, patient understanding, or safety.

Justice requires that telemedicine services be equitably accessible. Islamic perspectives on digital health ethics emphasize fairness and social responsibility, particularly in ensuring that vulnerable or underserved populations are not disadvantaged by digital disparities [17]. This aligns with broader principles of health justice and supports the development of inclusive, culturally sensitive virtual care systems. Therefore, integrating ethical principles into telemedicine requires an approach that upholds Islamic values, supports patient autonomy within family-centered contexts, and ensures safe, equitable practice. This provides a coherent ethical foundation for guiding telemedicine implementation in Saudi primary healthcare [16-18,21].

Barriers to implementation in Saudi primary care

Despite significant progress in digital health, multiple barriers continue to hinder the effective adoption of telemedicine in Saudi primary care. Broader evaluations of healthcare innovation highlight organizational readiness, workflow disruption, and limited stakeholder engagement as major obstacles to integrating new technologies, including virtual care [33]. These structural challenges can prevent primary care clinics from incorporating telemedicine smoothly into routine practice.

Studies involving Saudi healthcare practitioners identify additional implementation difficulties. Physicians frequently report technical problems, limited training, inconsistent connectivity, and uncertainty about clinical responsibilities during remote consultations [3,5]. Variation in digital literacy among providers can further impede their ability to conduct virtual assessments and maintain care quality [3]. Rapid telemedicine expansion during the COVID-19 pandemic also created workflow pressures and highlighted gaps in provider preparation [5].

Patient-related barriers also complicate implementation. Older adults often face multiple challenges, as previously discussed, which collectively reduce their engagement with telemedicine [14]. Systematic reviews of telehealth adoption in Saudi Arabia note similar issues among the general population, including low technological familiarity, concerns about care reliability, and a persistent preference for in-person visits [13].

System-level insights provide additional context. Evaluations of the national 937 virtual call center, while operating within a centralized model distinct from primary care clinics, demonstrate that sustaining high-quality telemedicine services at scale requires strong coordination, adequate staffing, and reliable communication pathways, offering system-level lessons relevant to primary care telemedicine planning [7]. These operational demands illustrate the importance of robust infrastructure for scalable telemedicine delivery. Improving technological readiness, provider training, patient digital literacy, and overall system capacity is vital for overcoming implementation challenges in telemedicine. Progress in these areas will enable the delivery of more dependable, equitable, and high-quality virtual care across Saudi primary healthcare.

Quality outcomes and patient safety in telemedicine

Telemedicine can enhance clinical outcomes, improve continuity of care, and increase patient access when supported by structured systems. Evidence shows that virtual care can facilitate timely intervention, reduce unnecessary in-person visits, and improve patient satisfaction across various settings [8]. Ensuring consistent quality, however, requires adherence to standardized protocols. Quality assurance studies emphasize the importance of identity verification, accurate documentation, appropriate triage, and timely follow-up to maintain safe and effective remote care [35]. These measures help prevent diagnostic errors and support reliable clinical decision-making. System-level evaluations similarly highlight that dependable digital infrastructure, interoperable systems, and well-designed workflows are essential for achieving high-quality telemedicine outcomes [44].

Patient safety remains a central concern. Reviews of virtual primary care indicate that structured assessment methods, emergency protocols, and contingency plans for technical issues are critical to mitigating risks associated with limited physical examination and potential communication barriers [36]. Clear guidelines for escalation and follow-up help ensure continuity of care during disruptions. When supported by strong infrastructure, standardized practices, and ongoing evaluation, telemedicine can function as a safe, effective, and reliable component of primary healthcare [8,35,36,44].

Physician workload and telemedicine intensity

Telemedicine can increase physician workload by expanding the volume and complexity of clinical tasks. Studies show that virtual care and patient-initiated messages often extend work beyond scheduled hours, contributing to higher administrative burden [45]. Qualitative research indicates that remote communication can blur professional boundaries, creating expectations for rapid responses and continuous availability [37]. Maintaining telehealth quality further requires detailed documentation, structured triage, and verification procedures, adding to clinicians’ responsibilities [35]. These factors collectively intensify workload, underscoring the need for clear guidelines and supportive systems to prevent strain and ensure sustainable virtual care [35,37,45].

Success factors for digital health implementation

Effective digital health implementation requires alignment between organizational readiness, technical capacity, and cultural context. Research on healthcare innovation shows that strong leadership, clear communication, and active stakeholder engagement are essential for integrating digital tools into clinical workflows [33]. When organizations coordinate implementation efforts and ensure that digital systems complement existing practices, telemedicine is more likely to be adopted consistently and used effectively.

Systematic reviews highlight that user-centered design and sustained institutional support are critical for long-term success [46]. Key factors include intuitive interfaces, dependable system performance, interoperability with existing health information systems, and continuous training for clinicians. These elements ensure that digital solutions are both functional and practical in everyday clinical settings.

Cultural adaptation is also crucial. Studies demonstrate that digital health technologies must reflect local norms, language, and communication preferences to achieve meaningful engagement [47,48]. Without cultural alignment, users may encounter barriers related to trust, usability, or comprehension. Successful adaptation requires iterative, participatory design processes that involve patients and clinicians throughout development [48].

Patient-centered telemedicine frameworks reinforce the importance of tailoring digital health tools to patient needs and values [21]. This includes clear communication, support for users with diverse levels of digital literacy, and mechanisms that incorporate patient feedback into system improvement. Such approaches strengthen usability and trust in virtual care. Successful telemedicine implementation requires coordinated improvements in technology readiness, provider training, patient digital skills, and system-wide support structures. Addressing these factors will enable more reliable, fair, and impactful virtual care across Saudi primary healthcare settings. [21,33,46-48].

Cultural adaptation of digital health technologies

Cultural adaptation is essential for ensuring that digital health technologies are accepted and effectively used in settings such as Saudi Arabia, where healthcare practices are shaped by Islamic ethical principles and family-centered decision-making norms [17,18]. These cultural expectations influence how patients interpret medical information, engage in virtual communication, and involve family members in care. Research from Saudi Arabia shows wide variation in patient preferences: some individuals expect strong family involvement, while others prefer more autonomous or physician-led approaches [18]. Studies across the Eastern Mediterranean Region similarly demonstrate that collective decision-making, hierarchical communication styles, and community-oriented values strongly influence engagement with digital health tools [20]. Telemedicine platforms must therefore accommodate diverse disclosure preferences and communication norms.

Global analyses highlight risks when digital interventions are deployed without cultural alignment. Challenges include mismatched communication styles, lack of sensitivity to local norms, and low usability when systems are designed around assumptions not shared by the target population [47]. Systematic reviews emphasize that meaningful cultural adaptation requires structured, iterative processes that assess user needs, involve community stakeholders, modify content and communication style, and refine systems based on feedback [48].

Integrating cultural adaptation into telemedicine design helps build trust and ensures accessibility. In Saudi Arabia, this includes aligning systems with Islamic ethical values, supporting family-inclusive decision-making when appropriate, and enabling clinicians to navigate varied patient preferences [17,18,20,47,48]. Such approaches strengthen user engagement and promote equitable, person-centered digital care.

Digital health equity framework

Digital health equity frameworks highlight that telemedicine can widen disparities if systems are not intentionally designed to address social and structural barriers [39,49]. Factors such as socioeconomic status, geography, and digital literacy strongly influence who benefits from virtual care [40]. Vulnerable groups, including low-income families, rural residents, and maternal-child populations, face reduced access due to limited connectivity and device availability [38,42]. Equity-focused frameworks recommend community co-design, culturally relevant interfaces, and infrastructure investments to ensure digital health initiatives are accessible and inclusive [39,49]. Integrating these principles helps prevent widening gaps and promotes fair, patient-centered telemedicine.

Regulatory and policy framework

The expansion of telemedicine requires strong regulatory and policy frameworks to ensure safe, secure, and ethically governed digital healthcare. Health information governance frameworks emphasize clarifying roles, setting data-handling standards, and defining accountability to protect patient information and maintain trust [25]. Globally, regulatory analyses identify challenges such as data privacy risks, cross-border data flows, and inconsistent legal protections, particularly when telemedicine relies on multiple platforms or third-party applications [26]. These issues highlight the need for unified standards, transparent data-sharing agreements, and robust enforcement mechanisms.

In Saudi Arabia, Vision 2030 has accelerated efforts to improve cybersecurity and digital governance in healthcare. National initiatives aim to strengthen security controls, enhance cyber resilience, and safeguard hospital infrastructures in recognition of the growing risks associated with expanded remote access and data transmission [30]. As telemedicine increasingly incorporates artificial intelligence, regulatory analyses stress the importance of AI-specific frameworks to address algorithmic vulnerabilities, potential manipulation, and emerging cyber threats. Clear standards for auditing, transparency, and responsible AI use are essential to ensure safety and prevent misuse [31].

International policy reviews further emphasize the need for clear definitions of virtual care modalities, licensure requirements, cross-jurisdictional practice rules, and reimbursement processes, as well as legal guidance on teleconsent and digital documentation [50]. Cybersecurity remains central to regulation, with blockchain technologies proposed as promising tools for enhancing data integrity and traceability across telemedicine systems [32].

Alignment with vision 2030

Saudi Arabia’s commitment to telemedicine is strongly shaped by the broader healthcare transformation agenda outlined in Vision 2030. This national framework identifies digital health as a strategic priority for improving the quality, accessibility, and efficiency of healthcare services across the Kingdom [1]. Vision 2030 initiatives aim to modernize service delivery, enhance system integration, and leverage technology to support patient-centered care, making telemedicine a central component of ongoing reform [1,2]. Advances in health informatics have played a critical role in operationalizing these goals. National strategies focus on improving interoperability, building unified digital platforms, and integrating electronic health records across healthcare institutions [2]. These efforts are designed to facilitate seamless communication between patients and providers, reduce fragmentation of care, and create a foundation for large-scale telemedicine deployment. As a result, digital tools are increasingly embedded within routine clinical workflows, supporting remote consultations, virtual triage services, and expanded access to primary care [2,13].

Cybersecurity and data protection have also become essential considerations in aligning telemedicine with Vision 2030 objectives. As digital transformation accelerates, national cybersecurity frameworks emphasize the need to safeguard hospital information systems, strengthen cyber readiness, and protect sensitive health data from emerging threats [30]. These measures are critical for ensuring that telemedicine services operate within secure and resilient infrastructures capable of supporting long-term digital innovation. Systematic reviews of telehealth adoption in Saudi Arabia highlight that Vision 2030 has accelerated both institutional readiness and public acceptance of digital care, though challenges related to usability, training, and patient digital literacy remain [13]. Addressing these barriers is an integral part of the national strategy to ensure equitable digital access and sustainable integration of telemedicine across diverse patient populations.

Overall, the alignment between telemedicine and Vision 2030 reflects a cohesive national vision that prioritizes digital innovation, system modernization, and patient-centered care. Strengthening digital infrastructure, enhancing cybersecurity, and improving workforce readiness remain key components for realizing the full potential of telemedicine as part of Saudi Arabia’s long-term healthcare transformation [1,2,13,30].

Practice implications

Effective integration of telemedicine into Saudi primary care requires strengthening clinician training, improving patient digital literacy, and establishing consistent protocols for remote consultations. Studies among Saudi physicians highlight the need for clearer guidance on clinical responsibilities, structured communication standards, and workflow adjustments to manage virtual care safely and efficiently [3,5]. Improving digital literacy is essential for reducing disparities in telemedicine access [6,14]. Quality and safety guidelines, including standardized triage, thorough documentation, and contingency plans for technical interruptions, support reliable clinical decision-making in virtual settings [35,36]. Additionally, increasing physician workload associated with telemedicine underscores the importance of workload management strategies and institutional support [45]. Patient-centered frameworks recommend routinely assessing patient preferences, communication needs, and family involvement to ensure that virtual care aligns with cultural expectations and ethical standards [21].

Limitations

This review has several limitations. Narrative reviews rely on selective integration of evidence and do not provide the structured rigor of systematic reviews, which may introduce interpretive bias. In the present review, such bias may arise from the defined search scope, inclusion criteria, and thematic synthesis approach, which may have resulted in greater emphasis on selected ethical domains-such as privacy, informed consent, and professional boundaries-and on regulatory and institutional perspectives most relevant to primary care telemedicine. This selective emphasis may influence how certain ethical challenges are foregrounded over others and may affect the applicability of some conclusions across all Saudi primary care contexts.

The rapid evolution of digital health technologies also means that regulatory and ethical developments may outpace published literature, limiting the currency of available evidence. Additionally, variations in study design, scope, and reporting across the included research complicate direct comparison of findings. Finally, while this review incorporates studies relevant to Saudi Arabia and comparable cultural contexts, some insights are drawn from broader international literature and may not fully capture all local nuances.

## Conclusions

Telemedicine is increasingly incorporated into healthcare delivery in Saudi Arabia and has demonstrated potential benefits in improving access to care, patient convenience, and service efficiency, particularly in urban settings and during periods of heightened system demand. However, the evidence synthesized in this review also highlights substantial and persistent challenges, including variability in digital literacy, inequities in access, concerns regarding informed consent and privacy, workload and workflow pressures, and inconsistent system readiness across primary care settings.

Addressing these ethical and operational issues, through clear and context-sensitive protocols, culturally appropriate communication strategies, clinician training, and targeted digital literacy initiatives, is essential to support safe and consistent telemedicine practice. With coordinated clinical, organizational, and policy-level efforts that acknowledge existing constraints and variability, telemedicine can continue to evolve as a component of patient-centered care within Saudi Arabia’s modernizing healthcare system.
